# Location Contexts of User Check-Ins to Model Urban Geo Life-Style Patterns

**DOI:** 10.1371/journal.pone.0124819

**Published:** 2015-05-13

**Authors:** Samiul Hasan, Satish V. Ukkusuri

**Affiliations:** 1 Land and Water Flagship, CSIRO, Melbourne, Victoria, Australia; 2 School of Civil Engineering, Purdue University, West Lafayette, Indiana, USA; Hangzhou Normal University, CHINA

## Abstract

Geo-location data from social media offers us information, in new ways, to understand people's attitudes and interests through their activity choices. In this paper, we explore the idea of inferring individual life-style patterns from activity-location choices revealed in social media. We present a model to understand life-style patterns using the contextual information (e. g. location categories) of user check-ins. Probabilistic topic models are developed to infer individual geo life-style patterns from two perspectives: i) to characterize the patterns of user interests to different types of places and ii) to characterize the patterns of user visits to different neighborhoods. The method is applied to a dataset of Foursquare check-ins of the users from New York City. The co-existence of several location contexts and the corresponding probabilities in a given pattern provide useful information about user interests and choices. It is found that geo life-style patterns have similar items—either nearby neighborhoods or similar location categories. The semantic and geographic proximity of the items in a pattern reflects the hidden regularity in user preferences and location choice behavior.

## Introduction

The recent introduction of the location-based services in smartphone-based social media applications allows people to share their activity related choices in their virtual social networks (e.g., Facebook Places, Foursquare). Such sharing occurs at the level of specific geo-location and time of where and when an individual participates in an activity. This vast amount of geo-location data offers us, in new ways, people’s attitudes and interests through their activity-location choices over a large number of users and over multiple months/years that was unimaginable before. In addition to location and timing, this data reveals our interests towards specific brands or types of locations for different activity purposes (e.g., Walmart, Target, Whole foods for grocery shopping). From this new information, we can understand human mobility behavior in a better way. We can infer users’ life-style choices from their activity-location choice patterns. However, while this new data is available, there are currently limited methodologies to infer *life-style choices* to characterize individual activity patterns important for understanding behavior. In this paper, using activity participation data shared in social media, we develop probabilistic models inferring individual interests to activity-locations and thereby obtain the life-style patterns of people living in an urban area.

The linkage between life-style and activity participation has been well recognized in activity-based travel behavior analysis [1–4] in the domain of transportation science. Individuals’ daily activity and travel patterns with longer-term choices of residential location, work place location and vehicle ownership are inter-related with each other and jointly define their lifestyle patterns [5]. Hence the concept of life-style choice provides a useful framework to study urban human activity and travel behaviors.

However, in the literature, there is no general consensus on a definition of life-style based on individual activity patterns. Life-style definitions vary from conceptual to operational ones. Most definitions belong to one of the two broader perspectives: a) life-style as behavioral patterns such as activity and time use patterns and b) life-style as a behavioral orientation [4]. The former approach views life-style as changing as an individual adapts to the surrounding environment while the later approach views life-style as an orientation which the individual wants to maintain by changing his or her actions. Changes in individual values, attitudes, and preferences may affect, in the long term, the individual’s life-style as orientation. Empirical studies have found evidence of the linkages between individual life-style choices and activity-travel behaviors. These behaviors include short-term choices, such as activity types and frequencies, travel distances, mode of travel [5–7], and more long-term choices, such as where to live and work [5] and what type of car to buy [8].

As location-based datasets from social media are becoming increasingly available, there is potential to characterize and infer user preferences from these datasets. Researchers have already been using these datasets to gather interesting insights on different aspects related to human mobility and activity choices. Studies from social science, computer science, and transportation science have used innovative ways to extract meaningful patterns with diverse applications. These studies include activity recognition [9], discovering mobility and activity choice behavior [10–16], classifying activity choice patterns [17], estimating urban travel demand and traffic flow [18–20], predicting next place to check-in [21], modeling the influence of friendship on mobility patterns [22], and detecting neighborhood boundaries [23].

With large sample size covering a long period (e.g., for a year) and providing locations and timings of individual activity participation, location-based datasets have further potential for activity-based modeling. However, a major challenge of using these datasets for activity behavior modeling is that individuals are recognized by only the identification numbers without any detailed information on socio-economic characteristics (e.g., income, age, race etc.). These attributes of the social media users are difficult to obtain. Without the socio-demographic attributes of the users, it is difficult to explain or to gain deeper insights from the observed patterns. We need to cluster the population based on a set of generic characteristics and correlate patterns with those characteristics providing a behavioral underpinning to different patterns observed from social media data. In this paper, we introduce the life-style concept giving a richer medium to understand the higher dimensions of activity patterns observed in social media.

We develop a clustering approach based on probabilistic topic models [24] to understand individual geo life-style patterns through their activity-location choices revealed in social media. We infer the life-style choice patterns using the location contexts of user check-ins. As location contexts, we use a combination of the names of the activity-locations, categories of the locations and the surrounding neighborhoods of the locations. We test our modeling approach to classify individual life-style patterns using a dataset of Foursquare check-ins (posted via Twitter) of 3256 users from New York City.

Our goal is to extract individual life-style patterns by modeling their interests towards specific types of restaurants, grocery or supermarkets, clothing stores etc., types of locations and neighborhoods. These life-style patterns will work as a bridge to understand the relationship between individual characteristics and their activity patterns. For instance, an individual’s interests towards more expensive types of restaurants may indicate his or her income level. On the other hand, a user’s visiting patterns to certain neighborhoods may reflect his or her local geographic context. Such geographic contexts can provide information on the socio-demographic characteristics such as income level, ethnicity etc. of the user.

Thus this analysis overcomes a major challenge of using social media data for behavior modeling in general. It helps to reconstruct individuals’ profiles although traditional socio-economic characteristics (e.g., income, race etc.) of those individuals are not observed. Thus the proposed method helps to classify individuals based on their geo life-style patterns. Such classification or segmentation of individuals will add some behavioral underpinnings to the observed patterns from social media data.

Previous studies [25, 26] used topic models to learn patterns from location-based social media data. For instance, Ferrari et al. [25] found city-level patterns that can identify hotspots in the city, and recognized aggregate-level behaviors recurring over time and space in the urban scenario. Similarly, Joseph et al. [26] used topic models for inferring aggregate patterns to identify groups of people with different types, communities and interests. However, we introduce the concept of life-style, for the first time, into the growing literature of activity-travel behavior analysis based on social media data and show its importance for behavioral modeling in general. Further, instead of inferring aggregate patterns based on topic models, we model user-specific patterns extending the topic modeling approach [24]. Such user-specific patterns recognize the user-level heterogeneity within the modeling framework. Our contributions are summarized as follows:

- We demonstrate that activity-location choice data from social media can be used to infer user life-style choices. We consider the categories of activity-locations and the neighborhoods representing the urban space semantics to infer user interests and spatial contexts.
- We show that topic models can be applied well to infer user life-style choices. We extend the topic modeling approach to infer user-specific patterns.
- We find that user interests to various activity-location choices have semantic and geographic proximity and we discuss the plausible reasons behind these proximities.

## Materials and Methods

### Dataset

We use check-in information posted via Twitter which allows its users to post their statuses from third-party check-in services (e.g. Foursquare). When foursquare users check-in to a place this status can be posted to their Twitter pages. We use a large-scale check-in dataset available from Cheng et al.[11]. The dataset contains check-ins from Feb, 25, 2010 to January, 20, 2011. After collecting the tweets, we pre-process the data where each data point is stored as a tuple with the following information: tweet(tweetID) = {userID, screen name, tweetID, date, location, text}

An example of a tweet with a check-in looks like (we replace the tweetID, userID, screen name and check-in ID with # so that the user cannot be identified):

tweet(#) = {#, #, #, Fri Jun 10 10:27:34 +0000 2011, 40.7529422, -73.9780177, “I’m at Central Cafe & Deli (16 Vanderbilt Ave., New York) http://4sq.com/#”}

**Fig 1:**
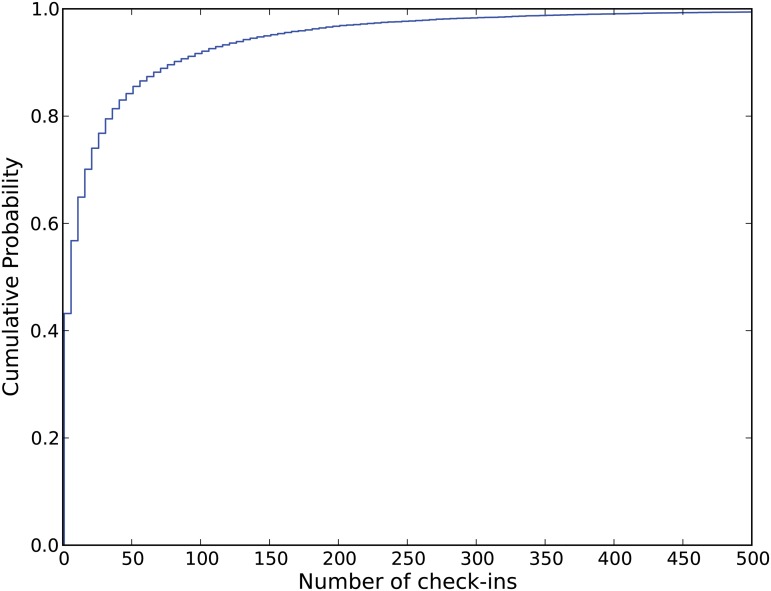
Check-in distribution.

After collecting the original dataset we select a subset of all the observations within New York City. We create a boundary region for New York City and keep all the check-in observations within that region. After doing this the New York dataset has 20606 users. Descriptive statistics on the number of check-in activities include: average = 33.03; min = 1; max = 1010; and standard deviation = 77.82. Fig 1 shows the cumulative distribution of the check-in activities. To find individual life-style patterns, we study only the geo-active users having more than 50 check-ins. Basic information about the dataset is given in Table 1.

**Table 1:** Dataset details.

Original dataset	
Number of users	20606
Number of check-ins	680564
Study sample	
Number of geo-active users	3256
Number of check-ins from geo-active users	504000

### Modeling Framework

User check-ins can be enriched with additional location contexts. Such contexts include the name of the place where the user has checked-in, the category of the place and the neighborhood of the area. Each of the collected tweets with a check-in from Foursquare has a short link. We run a query using the Foursquare API with the short link as an input and collect the detailed check-in information including the name and category of location where the user has checked-in. Thus we enrich the location contexts of each check-in with names and/or category of the location and the neighborhood of the place to model individual life-style patterns. This location category information can also be used to label the category of the activity for each check-in and consequently be used for classifying user activity patterns.

**Fig 2:**
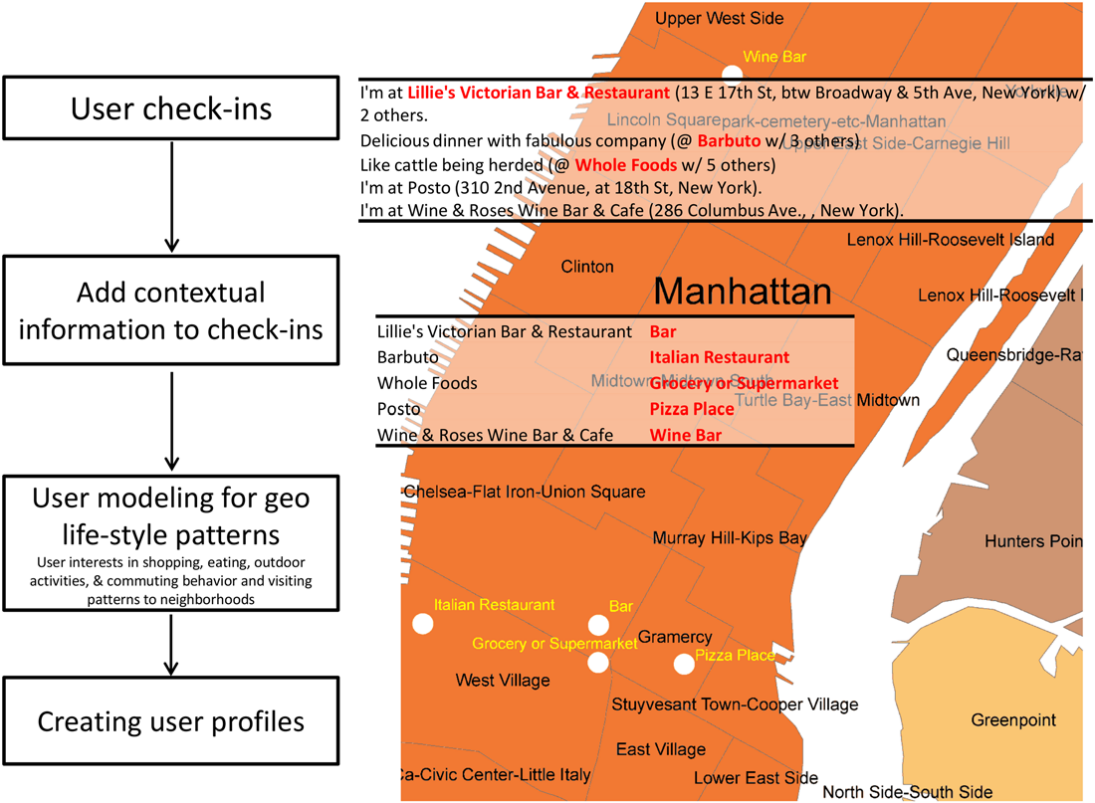
Components of the proposed approach to enrich the location contexts of user check-ins and use it for modeling geo life-style patterns.

Fig 2 illustrates the components of our modeling approach to infer life-style patterns from the rich semantics of user check-ins. At first user check-ins are collected. The check-ins contain the names and addresses of the activity-locations that the user has visited. In the next stage, additional contextual information is added to the check-in observations. For example, based on the name of a place, its category can be extracted from a database. In this study, we collect the categories of the activity-locations from Foursquare venue category database. Similarly, from the address of a location we can find the neighborhood of the location. In the next stage, location contexts of user check-ins can be modeled to classify user life-style patterns.

In the present work, as location contexts, we use a combination of the names of the activity-locations, categories of the locations and the surrounding neighborhoods of the locations. We represent individual life-style patterns from two perspectives: i) through the patterns of users’ interests in different places and ii) through the patterns of users’ visits to different neighborhoods.

### Problem Description

Inferring life-style patterns involves finding the complex patterns of individual interests from his/her everyday activity-locations choices. Life-style patterns can be represented as a distribution of location contexts where each context can be represented by the name, category and/or neighborhood of the location. Geo life-style pattern inference problem can be defined as: given the location contexts for *n* visits as *c*_1_, *c*_2_,…, *c*_*n*_ of user *u*, determine the *K* latent life-style patterns through *ϕ*_*k*_, *k* ∈ 1, 2,.., *K* where each life-style pattern *ϕ*_*k*_ is a distribution of location contexts.

### Model Description

**Fig 3:**
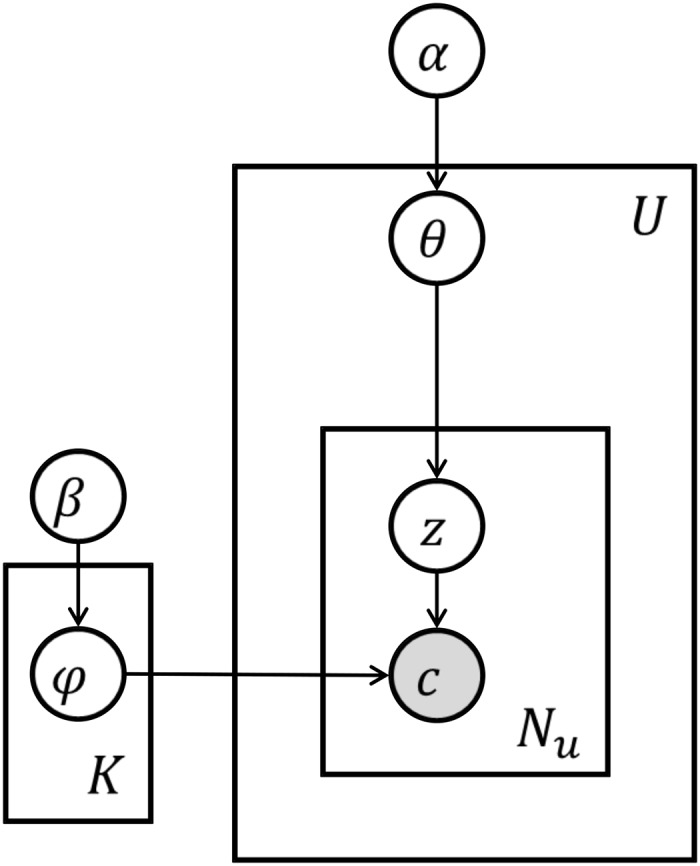
Topic model of geo life-style pattern inference. White circles represent random variables, shaded circles represent observed variables, rectangles represent the repetitiveness of the data, and arrows represent the dependency among the entities.

We present the topic model of individual geo life-style patterns assuming that each user’s life-style is a distribution of patterns where each pattern is a distribution of location contexts. A graphical representation of the model is shown in Fig 3. The modeling framework extends the topic modeling approach [24].

The probabilistic generative process for the model is summarized as following:

1. For each life-style pattern *k* ∈ 1, 2,…, *K*, select a distribution over location contexts *ϕ* ^(*k*)^ ∼ *Dirichlet*(*β*)
2. For each user *u* ∈ 1, 2,…, *U*,
  - Select a distribution over life-style patterns *θ* ^(*u*)^ ∼ *Dirichlet*(*α*)
  - For each location context *i*,
    - Select a pattern *z*_*i*_ ∼ *Multinomial*(*θ* ^(*u*)^); *z*_*i*_ ∈ 1, 2,…, *K*
    - From life-style pattern *z*_*i*_, select a location context
*c*_*i*_ ∼ *Multinomial*(*ϕ* ^(*z*_*i*_)^);*c*_*i*_ ∈ 1, 2,…, *C*

Given *U* users’ location contexts, *K* life-style patterns over *C* unique location-contexts, the objectives of the inference of life-style pattern extraction are to:

1. find the probability of a location-context *c* given each pattern *k*, $P(c|z=k)=\phi_{k}^{c}$ where *P*(*c*|*z* = *k*) is represented with *K* multinomial distributions *ϕ* over location contexts of size *C*.
2. find the probability of a pattern *k* for a location context in the location visits of user *u*, $P(z=k|u)=\theta_{u}^{k}$. Here *P*(*z*|*u*) is represented with *U* multinomial distributions *θ* over *K* life-style patterns.

The above model views a life-style pattern as a probability distribution over location contexts and user life-style as a mixture of these patterns. From *K* life-style patterns, the probability of *i* ^*th*^ location context for a given user *u* is:

$$\begin{array}{c} P\left(c_{i}|u\right)=\sum_{j=1}^{K}P\left(c_{i}|z_{i}=j\right)P\left(z_{i}=j|u\right) \end{array}$$ (1)

where *z*_*i*_ is the latent variable indicating the pattern from which the *i* ^*th*^ location context is drawn, *P*(*c*_*i*_|*z*_*i*_ = *j*) is the probability of the location context *c*_*i*_ under the *j* ^*th*^ pattern and *P*(*z*_*i*_ = *j*|*u*) is the probability of choosing a location context from pattern *j* in the location visits of user *u*. Intuitively, *P*(*c*|*z*) determines the importance of a location context to a pattern and *P*(*z*|*u*) determines the prevalence of the patterns in user *u*’s location choices.

### Parameter Estimation

In practice, there are various approximate techniques for estimating the parameters of this model [24, 27]. We use the Gibbs sampling approach proposed by [27]. Details of the Gibbs sampling approach can be found in [27].

The joint distribution *P*(**c**, **z**) can be written as

$$\begin{array}{c} P(c,z)=P(c|z)P(z) \end{array}$$ (2)

First term can be found as:

$$\begin{array}{c} P\left(c|z\right)={\left(\frac{\Gamma (C\beta )}{\Gamma {\left(\beta \right)}^{C}}\right)}^{K}\prod_{k=1}^{K}\frac{\prod_{c}\Gamma (n_{k}^{c}+\beta )}{\Gamma (n_{k}^{\left(.\right)}+C\beta )} \end{array}$$ (3)

where $n_{k}^{c}$ is the number of times location context *c* is assigned to pattern *k* and $n_{k}^{\left(.\right)}=\sum_{c=1}^{C}n_{k}^{c}$

The second term can be written as:

$$\begin{array}{c} P\left(z\right)={\left(\frac{\Gamma (K\alpha )}{\Gamma {\left(\alpha \right)}^{K}}\right)}^{U}\prod_{u=1}^{U}\frac{\prod_{k}\Gamma (n_{u}^{k}+\alpha )}{\Gamma (n_{u}^{\left(.\right)}+K\alpha )} \end{array}$$ (4)

where $n_{u}^{k}$ is the number of times a location context from user *u* is assigned to pattern *k* and $n_{u}^{\left(.\right)}=\sum_{k=1}^{K}n_{u}^{k}$

For applying the Gibbs sampling approach, a pattern can be assigned using the following conditional distribution:

$$\begin{array}{c} P\left(z_{i}=k|z_{-i},c,u\right)\propto \frac{n_{-i,k}^{c_{i}}+\beta }{n_{-i,k}^{\left(.\right)}+C\beta }\frac{n_{-i,u_{i}}^{k}+\alpha }{n_{-i,u_{i}}^{\left(.\right)}+K\alpha } \end{array}$$ (5)

where *n*_−*i*_ is the count excluding the current pattern assignment of *z*_*i*_. Intuitively, Eq 5 expresses two ratios; the first ratio expresses the probability of location context *c*_*i*_ in pattern *k* and the second ratio expresses the probability of pattern *k* in the life-style patterns of user *u*. Finally model parameters can be computed as:

$$\begin{array}{ccc} {\hat{\Phi }}_{k}^{c} & = & \frac{n_{k}^{c}+\beta }{n_{k}^{\left(.\right)}+C\beta } \end{array}$$ (6)

$$\begin{array}{ccc} {\hat{\theta }}_{u}^{k} & = & \frac{n_{u}^{k}+\alpha }{n_{u}^{\left(.\right)}+K\alpha } \end{array}$$ (7)

## Results

### Model Selection

For model selection, we first run our algorithm for different number of life-style patterns (*K*) and compute a metric called perplexity to measure how well the model can predict the unseen data for each run. We then select the optimal number of patterns based on perplexity values. Perplexity of a test dataset, a set of location contexts {**c**_*u*_} for *u* ∈ *U* ^*test*^, given a model ℳ is defined as

$$\begin{array}{c} Perplexity=\exp[-\frac{\sum_{u=1}^{U}\log p(c_{u}|𝓜)}{\sum N_{u}}] \end{array}$$ (8)

**Fig 4:**
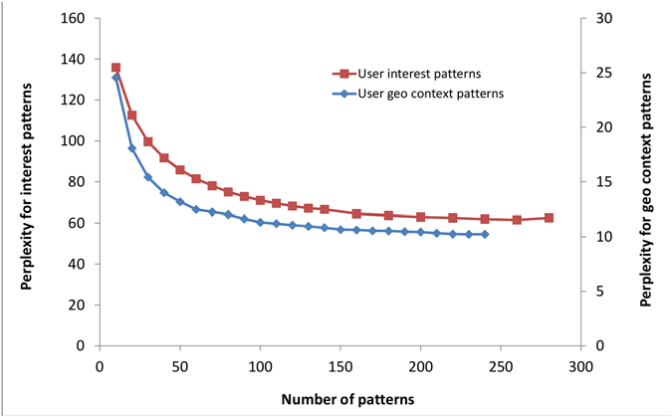
Perplexity versus the number of life-style patterns.

Where *N*_*u*_ is the number of location contexts of user *u* and *p*(**c**_*u*_|ℳ) can be computed using Eq 1. For estimating perplexity values we randomly split the dataset with 90% of the users in training set and rest 10% in the test set; estimate the life-style pattern model on the training dataset; and compute the perplexity values on the test dataset. Fig 4 shows the results for perplexity measurements for life-style pattern model. A lower perplexity value indicates better performance of the model. It is found that with the increase of the number of life-style patterns the perplexity value reduces however there is no significant improvement beyond a certain number of patterns.

### Inferring User Interests

To model user interests to different types of places such as restaurants, stores, grocery shops etc., we use a set of location contexts which mainly include the location categories. For selected location categories, we represent the location contexts by the names of the places. A list of the examples of location contexts used for modeling user interests is presented in Table 2.

**Table 2:** Examples of location categories used and the list of the categories for which names are used.

Few of the location categories used	Location categories for which names are used
American Restaurant	Burrito Place
Asian Restaurant	Burger Joint
Bar	Clothing Store
Baseball Stadium	Coffee Shop
Boat or Ferry	Cosmetics Shop
Bookstore	Department Store
Bridge	Drugstore or Pharmacy
Building	Electronics Store
College Quad	Fast Food Restaurant
Cupcake Shop	Furniture or Home Store
Dessert Shop	Grocery or Supermarket
Diner	Women’s Store
Donut Shop	Gift Shop
Event Space	Kids Store
Falafel Restaurant	Mall
Food Truck	Sandwich Place
French Restaurant	Toy or Game Store
Gastropub	Men’s Store
General College & University	Wings Joint
Harbor or Marina	
Hardware Store	
Home	
Hospital	
Hotel	
Ice Cream Shop	
Italian Restaurant	
Juice Bar	
Karaoke Bar	
Latin American Restaurant	
Lounge	
Mexican Restaurant	
Middle Eastern Restaurant	
Miscellaneous Shop	
Movie Theater	

Based on the perplexity values, we select *K* = 100 for estimating the model for user interests to different activity-location contexts. Table 3 presents a few patterns estimated and the probability of the top 10 location-contexts for each pattern reported.

**Table 3:** Results of user interests pattern model.

**Pattern1**	**0.0072**	**Pattern2**	**0.0079**	**Pattern3**	**0.0083**
Activity Location	Prob.	Activity Location	Prob.	Activity Location	Prob.
Residential Building	0.3315	Doctor’s Office	0.1836	Food Truck	0.5114
Beach	0.1642	Post Office	0.1655	Indian Restaurant	0.2006
Courthouse	0.0827	Light Rail	0.1122	Shake Shack	0.0217
Park	0.0529	Movie Theater	0.0718	Asian Restaurant	0.0154
Theme Park	0.0355	Pathmark	0.0356	Temple	0.0147
Levi’s	0.0226	Grocery or Supermarket	0.0341	Restaurant	0.0137
Government Building	0.0210	Walmart	0.0315	FIKA espresso bar	0.0130
Hookah Bar	0.0190	Ice Cream Shop	0.0282	4food	0.0109
American Restaurant	0.0182	Diner	0.0271	Brewery	0.0102
Multiplex	0.0158	Parking	0.0182	Dessert Shop	0.0081
**Pattern4**	**0.0114**	**Pattern6**	**0.0105**	**Pattern10**	**0.0217**
Activity Location	Prob.	Activity Location	Prob.	Activity Location	Prob.
Pub	0.6889	Korean Restaurant	0.1999	Train Station	0.9410
Bar	0.1501	Ramen or Noodle House	0.1934	Train	0.0031
Sports Bar	0.0432	Vietnamese Restaurant	0.1054	Belmont Buy Any Drug	0.0023
Lounge	0.0063	Asian Restaurant	0.0728	Newport Centre Mall	0.0021
A&P	0.0060	Japanese Restaurant	0.0579	Cajun Restaurant	0.0011
Rogo	0.0034	Chinese Restaurant	0.0470	Historic Site	0.0011
Pool Hall	0.0032	Dumpling Restaurant	0.0413	My Way Cup	0.0011
Harry’s Burrito	0.0032	Malaysian Restaurant	0.0225	Light Rail	0.0010
Dive Bar	0.0026	Indian Restaurant	0.0189	Captain Caf	0.0010
Apple Store	0.0021	Dessert Shop	0.0183	Transit	0.0009
**Pattern17**	**0.0085**	**Pattern20**	**0.0061**	**Pattern23**	**0.0095**
Activity Location	Prob.	Activity Location	Prob.	Activity Location	Prob.
Sushi Restaurant	0.4740	College & University	0.2502	Target	0.1950
Japanese Restaurant	0.3324	College Dorm	0.2121	McDonald’s	0.1573
Caf	0.0151	College Building	0.0661	Hookah Bar	0.0921
Indian Restaurant	0.0096	Synagogue	0.0531	Bes	0.0621
Chinese Restaurant	0.0086	Student Center	0.0451	Burger King	0.0451
Sunrise Mart	0.0062	Basketball Court	0.0335	Fast Food Restaurant	0.0420
Brewery	0.0048	Harbor or Marina	0.0196	Wendy’s	0.0340
Cemetery	0.0038	Park	0.0186	Video Game Store	0.0331
Seafood Restaurant	0.0035	Multiplex	0.0159	Walgreens	0.0309
Spanish Restaurant	0.0031	College Auditorium	0.0135	Toys R Us	0.0288
**Pattern36**	**0.0101**	**Pattern49**	**0.0086**	**Pattern56**	**0.0076**
Activity Location	Prob.	Activity Location	Prob.	Activity Location	Prob.
Highway or Road	0.6841	Baseball Stadium	0.4809	Seafood Restaurant	0.2439
General Travel	0.1416	Stadium	0.2119	Trader Joe’s	0.1757
Bridge	0.0378	Football Stadium	0.1043	Italian Restaurant	0.0850
Waldbaums	0.0088	Sports Bar	0.0294	American Restaurant	0.0732
Stop & Shop	0.0050	Basketball Stadium	0.0127	Grey Dog	0.0568
Hot Spring	0.0050	Baseball Field	0.0106	French Restaurant	0.0317
Chinese Restaurant	0.0047	Five Guys Burgers	0.0089	Mud	0.0309
Bob’s Discount Furniture	0.0032	Racetrack	0.0062	Kaffe 1668	0.0237
Neighborhood	0.0024	Plane	0.0062	Pizza Place	0.0130
Jersey Gardens	0.0024	Basketball Court	0.0035	Asian Restaurant	0.0122
**Pattern41**	**0.0136**	**Pattern62**	**0.0093**	**Pattern70**	**0.0082**
Activity Location	Prob.	Activity Location	Prob.	Activity Location	Prob.
Airport	0.4956	Bus Line	0.5598	Chinese Restaurant	0.4795
Airport Terminal	0.3586	Subway	0.0822	Asian Restaurant	0.2854
Airport Gate	0.0289	Home	0.0496	Energy Kitchen	0.0352
Airport Tram	0.0175	Bus Station	0.0364	IKEA	0.0107
Plane	0.0175	Train Station	0.0335	Dim Sum Restaurant	0.0103
Airport Lounge	0.0106	Playground	0.0174	Zaro’s Bakery	0.0103
Government Building	0.0033	McDonald’s	0.0139	Apple Store	0.0100
New American Restaurant	0.0016	Train	0.0133	Italian Restaurant	0.0071
Candy Store	0.0013	PC Richard & Son	0.0124	Pizza Place	0.0064
Hotel	0.0011	Transit	0.0089	Cupcake Shop	0.0043

We observe that the activity locations in a given pattern have semantic similarity. For instance, all the locations in pattern 6 are restaurants. Top three locations in pattern 36 are related to highways. Most of the locations in pattern 62 are related to bus or train stations. Top five locations in pattern 49 are either stadiums or sports bars. From this semantic proximity, we can easily label these patterns and infer the latent interests and characteristics of the users contributing to these patterns. Interesting insights found from the results are as follows:

- Patterns 1 and 2 represent home, work and maintenance related activity locations.
- Patterns 3, 6, 17, 56 and 70 indicate users’ choices of restaurants and their eating behaviors.
- Pattern 20 represents the activity locations related to a college or university student.
- Pattern 49 represents user interests towards sports related events.
- Patterns 10, 36, 41 and 62 indicate the modal choice of the users. For instance, patterns 10 and 62 consist of the location contexts related to the transit riders and pattern 36 represents the location contexts related to a car user.

The co-existence of several location contexts and the corresponding probabilities in a given pattern provide useful information. For instance, pattern 6 has all the Eastern Asian restaurants as top location contexts indicating greater user preferences to the foods originated from this region. In pattern 56, we find that those who go to seafood restaurants have higher likelihood to go to Trader Joe’s for grocery shopping. Similar inferences can be drawn from other patterns.

**Fig 5:**
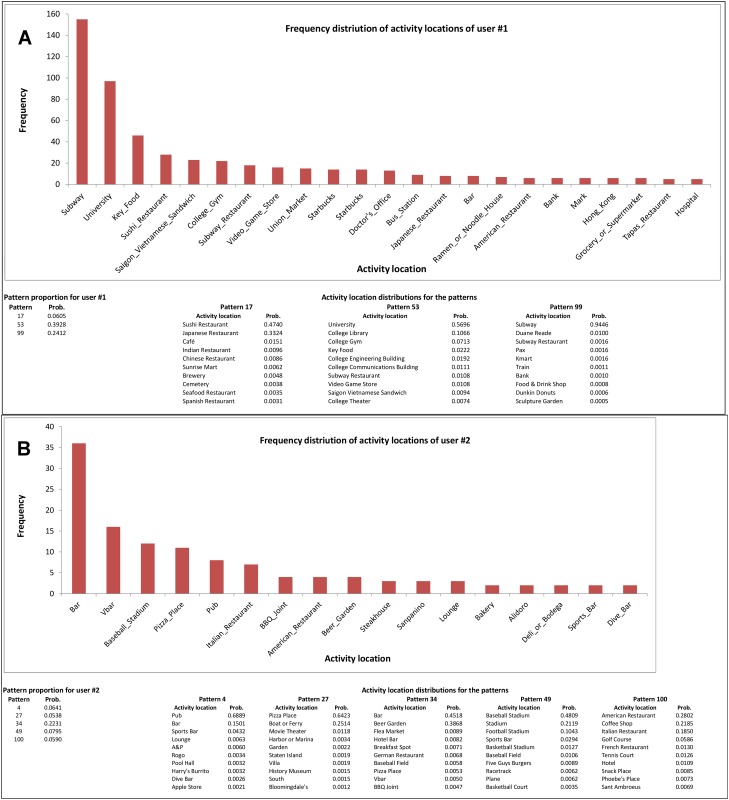
The distribution of location contexts of a user, the pattern proportion and the activity location distributions for the corresponding patterns. (A)User 1 having few dominating activity location choice patterns. For the pattern proportion of the user, only the patterns with probabilities not less than 0.05 are shown. For the activity location distribution, only the locations with frequencies not less than five are presented; the data contains 617 check-ins of the user. (B) User 2 having a diverse activity location choice patterns. For the pattern proportion of the user, only the patterns with probabilities not less than 0.05 are shown. For the activity location distribution, only the locations with frequencies not less than two are presented; the data contains 145 check-ins of the user.

To explore the relationship among the patterns and a user’s location choices, we report the distribution of location contexts of a user, his or her top most pattern proportions (*P*(*z*|*u*) ≥ 0.05) and the corresponding pattern descriptions (Fig 5). We choose two users, one with only few dominating patterns and another one with more diverse activity location choice patterns. These examples demonstrate how a user’s location choices can be modeled as a mixture of patterns as the user has chosen several location categories that belong to a few specific patterns. User 1 has a high degree of regularity in activity location choices as indicated by the lower number of dominating patterns, while user 2 has chosen a wider range of activity locations as indicated by the higher number of dominating patterns.

### Inferring User Geo Contexts

To model users’ visits to specific parts of the city and infer their life-style patterns from it, we use a set of neighborhoods to represent their location contexts. We collect the information of 195 neighborhoods in 5 boroughs of New York City [28] and label the coordinate for each check-in with the name of the corresponding neighborhood. Using the names of these neighborhoods as location contexts, we run our algorithm. Based on the perplexity values we select *K* = 50 for estimating the model parameters.

**Fig 6:**
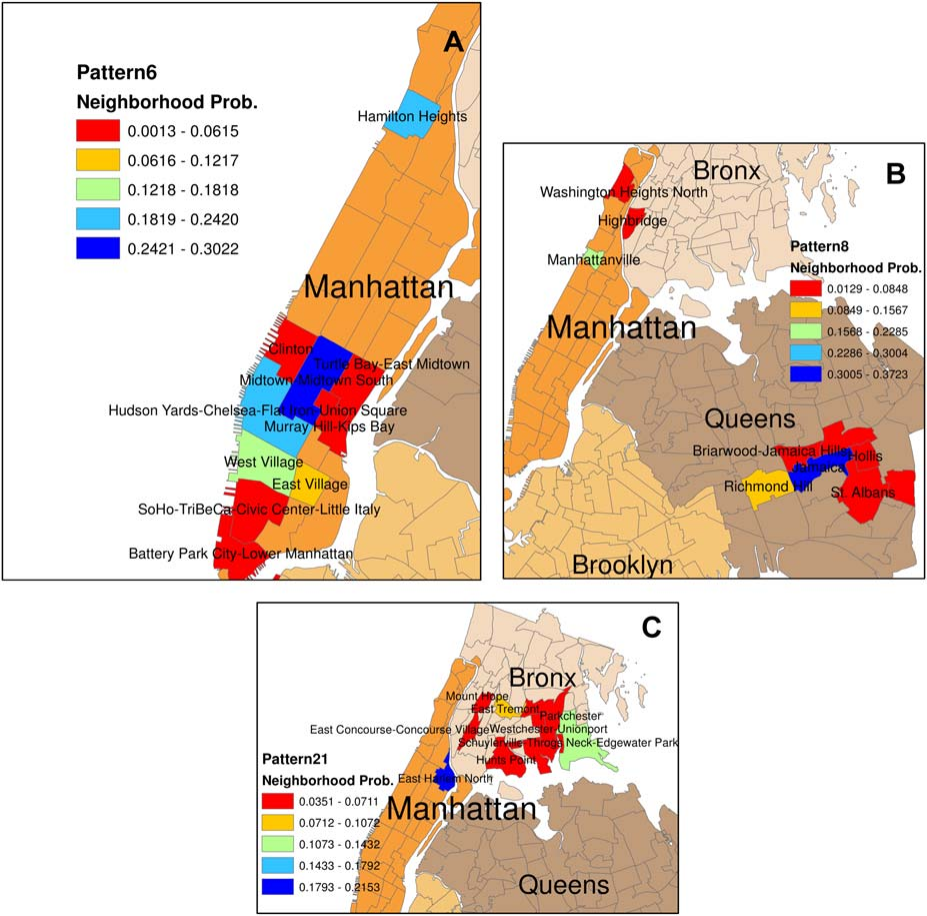
Geo context patterns in New York City. (A)Pattern 6 (B) Pattern 8 (C)Pattern 21

Table 4 presents a few of the patterns estimated and the probability of the top 10 neighborhoods for each pattern reported. To understand the spatial meaning of the patterns, we present few patterns in a map of New York City (see Fig 6). Each pattern reveals the predominant neighborhoods based on the check-in information from the data. One of the major findings from these patterns are the geographic proximity among the neighborhoods in each pattern. Even though we have not used the information on the spatial proximity of the neighborhoods as a priori knowledge, the hidden structure is revealed by the patterns of activity-location choices of the users. This means that individuals prefer nearby locations for different activity purposes. Using the pattern proportions for each user, we can infer his or her local neighborhood. Such information might be crucial to reconstruct a user’s profile.

**Table 4:** Results of user geo context pattern model.

**Pattern1**	**0.0135**	**Pattern2**	**0.0305**
Neighborhood	Prob.	Neighborhood	Prob.
Prospect Heights	0.3145	Battery Park City-Lower Manhattan	0.9292
park-cemetery-etc-Brooklyn	0.2866	East Flushing	0.0679
Hudson Yards-Chelsea-Flat Iron-Union Square	0.1194	Clinton	0.0005
West Village	0.1147	West New Brighton-New Brighton-St. George	0.0004
SoHo-TriBeCa-Civic Center-Little Italy	0.1119	Far Rockaway-Bayswater	0.0002
East Village	0.0169	Midtown-Midtown South	0.0002
Fort Greene	0.0146	Erasmus	0.0002
Chinatown	0.0095	Pelham Bay-Country Club-City Island	0.0002
DUMBO-Vinegar Hill-Downtown Brooklyn-Boerum Hill	0.0043	East Harlem South	0.0001
Clinton	0.0008	Williamsburg	0.0001
**Pattern4**	**0.0133**	**Pattern6**	**0.0138**
Neighborhood	Prob.	Neighborhood	Prob.
Forest Hills	0.3127	Midtown-Midtown South	0.3022
East Harlem South	0.1784	Hamilton Heights	0.1934
Glendale	0.1366	Hudson Yards-Chelsea-Flat Iron-Union Square	0.1893
Middle Village	0.1267	West Village	0.1349
Kew Gardens	0.1038	East Village	0.0876
Maspeth	0.0712	Clinton	0.0475
park-cemetery-etc-Queens	0.0247	SoHo-TriBeCa-Civic Center-Little Italy	0.0316
Rego Park	0.0199	Turtle Bay-East Midtown	0.0043
Lindenwood-Howard Beach	0.0146	Battery Park City-Lower Manhattan	0.0015
Ridgewood	0.0017	Murray Hill-Kips Bay	0.0013
**Pattern7**	**0.0103**	**Pattern8**	**0.0103**
Neighborhood	Prob.	Neighborhood	Prob.
Washington Heights South	0.3472	Jamaica	0.3723
Seagate-Coney Island	0.2169	Manhattanville	0.2139
Hudson Yards-Chelsea-Flat Iron-Union Square	0.1320	Richmond Hill	0.1017
SoHo-TriBeCa-Civic Center-Little Italy	0.1067	Highbridge	0.0784
West Village	0.0918	Briarwood-Jamaica Hills	0.0752
Midtown-Midtown South	0.0646	St. Albans	0.0663
East Village	0.0210	Jamaica Estates-Holliswood	0.0250
Manhattanville	0.0062	Washington Heights North	0.0198
West Brighton	0.0062	Hollis	0.0129
park-cemetery-etc-Manhattan	0.0011	Cambria Heights	0.0129
**Pattern10**	**0.0310**	**Pattern11**	**0.0128**
Neighborhood	Prob.	Neighborhood	Prob.
DUMBO-Vinegar Hill-Downtown Brooklyn-Boerum Hill	0.5179	Flatbush	0.1946
Carroll Gardens-Columbia Street-Red Hook	0.2996	Prospect Lefferts Gardens-Wingate	0.1832
Brooklyn Heights-Cobble Hill	0.1802	Brownsville	0.1727
Fort Greene	0.0002	Canarsie	0.1052
North Side-South Side	0.0002	East Flatbush-Farragut	0.0603
Manhattanville	0.0002	Erasmus	0.0598
Hudson Yards-Chelsea-Flat Iron-Union Square	0.0001	Crown Heights South	0.0553
Flatbush	0.0001	Crown Heights North	0.0529
Murray Hill	0.0001	Flatlands	0.0342
Forest Hills	0.0001	Rugby-Remsen Village	0.0235
**Pattern14**	**0.0126**	**Pattern21**	**0.0133**
Neighborhood	Prob.	Neighborhood	Prob.
Midtown-Midtown South	0.3743	East Harlem North	0.2153
park-cemetery-etc-Queens	0.3176	Schuylerville-Throgs Neck-Edgewater Park	0.1158
Woodside	0.1506	East Tremont	0.0965
Hudson Yards-Chelsea-Flat Iron-Union Square	0.0805	Van Nest-Morris Park-Westchester Square	0.0649
Queens Village	0.0599	Soundview-Castle Hill-Clason Point-Harding Park	0.0586
Corona	0.0048	Westchester-Unionport	0.0509
Airport	0.0041	Parkchester	0.0406
park-cemetery-etc-Manhattan	0.0037	East Concourse-Concourse Village	0.0404
Lincoln Square	0.0005	Hunts Point	0.0351
East Williamsburg	0.0004	Mount Hope	0.0351
**Pattern23**	**0.0176**	**Pattern41**	**0.0124**
Neighborhood	Prob.	Neighborhood	Prob.
New Springville-Bloomfield-Travis	0.1629	Jackson Heights	0.1970
Stapleton-Rosebank	0.1177	Rego Park	0.0967
Westerleigh	0.1077	Jamaica Estates-Holliswood	0.0700
West New Brighton-New Brighton-St. George	0.0965	Woodhaven	0.0620
Todt Hill-Emerson Hill-Heartland Village-Lighthouse Hill	0.0732	South Jamaica	0.0558
Charleston-Richmond Valley-Tottenville	0.0649	Bellerose	0.0556
Old Town-Dongan Hills-South Beach	0.0605	Springfield Gardens South-Brookville	0.0533
Great Kills	0.0546	Rosedale	0.0495
New Dorp-Midland Beach	0.0469	East Elmhurst	0.0492
Mariner’s Harbor-Arlington-Port Ivory-Graniteville	0.0407	Ozone Park	0.0488

## Discussion

This work demonstrates the use of social media data to obtain deeper insights into user life-styles from their activity-location choices. We present a model to infer geo life-style patterns based on contextual information available from check-in observations. These patterns reveal the hidden structure in user interests explaining their behavior. User interests to specific types of shopping places, restaurants, traveling mode, sporting events, and so on can reveal important information on their life-style choices. Using the names of the neighborhoods visited, we also model user geo life-style patterns indicating their geographic contexts. Inferring geo life-style patterns has powerful applications from explaining the observed patterns in social media to inferring user socio-economic characteristics.

Geo life-style patterns provide several valuable insights. For instance, a given pattern has similar items- either nearby neighborhoods or similar location categories. In the 100 patterns extracted to infer user interests, location contexts in the same pattern have semantic similarity. While in the 50 patterns extracted to infer user geo contexts, neighborhoods in the same pattern have geographic proximity. Why do locations with similar characteristics or nearby neighborhoods are more likely to appear in the same pattern?

The semantic proximity of the items in a given pattern suggests the hidden regularity in user preferences and interests. People are more likely to visit only a few similar types of places. The modality of user preferences to a few location contexts is picked up by the patterns. For instance, in Fig 5A, we find that a user having check-ins from Sushi restaurants has also check-ins from Japanese restaurants. One can argue that this phenomenon is merely due to the semantic biases in user check-in behavior. However, this may not be the case. We observe from the patterns that the purposes behind visiting the locations are mostly discretionary ones- representing infrequent visits. Regular check-in users are more likely to check-in from most of those infrequent visited locations- not from only a few of those location categories.

The geographic proximity among the neighborhoods in a given geo context pattern implies the geographic constraints faced by the users. There are costs, related to time and money, involved to travel from one place to another. The further a place is, the more one has to spend to travel to that place. Thus for different activity purposes, people are more likely to visit a few neighborhoods near to where they live or work or have social connections. This suggests a high degree of regularity in user location choices for participating in different activities. Similarly, in a previous study [29] discovering mobility patterns from mobile phone data, it was found that people spend most of their time to a few locations.

These findings have remarkable implications in activity-based travel behavior modeling. Researchers have been developing complicated choice models to predict individual activity-location choice behavior without considering the latent interests and spatial contexts of the users. One of the major challenges of estimating these choice models is the huge size of the choice set. To avoid this problem, typically a smaller choice set is generated at random. Our analysis indicates that the user interest patterns to activity-locations and the geo context patterns can give us an alternative and more realistic way to predict user activity-location choice behavior.

Based on their life-style choices, we can segment the study sample and correlate the observed activity behavior patterns with their life-style choices. Thus this analysis provides a behavioral underpinning to mobility and activity behavior observations [11, 12] based on social media data. This life-style concept may also provide a richer medium to understand other dimensions of behavioral patterns observed in social media. For instance, social media data has been used to observe various behavioral aspects in areas including marketing [30, 31], social network analysis [22], urban planning [23, 32], and health monitoring [33]. Such analyses can be enriched or extended to include life-style choices providing valuable explanation to the observed behavior.

To explain the behavioral patterns, a more traditional approach is to use the socio-demographic attributes of the users. Typically socio-demographic characteristics are used to segment a study sample and representative behavioral patterns are derived for these segmentation bases. Then the overall population is segmented based on similar characteristics available from census data and the representative patterns are assumed for the population segments. This step is needed to scale up from a survey sample to population behavior (e.g., estimate the travel demand for a city). However, these attributes of the social media users are difficult to obtain. Our approach can help to infer those socio-demographic characteristics of the study sample. Using the geo context patterns we next present an idea on how to construct user profiles.

Our objective is to create the socio-demographic profiles of the users based on their geo life-style patterns, or in simple words based on where they go. We may argue that people visiting the same neighborhood frequently are more likely to have similar socio-demographic characteristics and an individual’s geo context patterns can identify his or her local neighborhood. Similar approaches, popularly known as Geodemographics, have been used in marketing before [34].

The results from geo context patterns provide a distribution of patterns for each user. This distribution can be treated as a feature vector for each user. These distributions of geo context patterns can be used to assign each user a particular neighborhood. We can then use the average socio-demographic characteristics of this neighborhood to construct the user’s profile. For instance, if we are interested to infer user income level, the median household income of the neighborhood can be used as the income level for the user. We can assign each user a neighborhood and use the median income for the neighborhood as the income level of the user. In this way we can derive the income distribution of the users.

However, this approach needs to be carefully implemented addressing a number of questions. For instance, how can we choose a dominating neighborhood for a user? Which average socio-demographic characteristics of a neighborhood would be appropriate to be assigned to a user? Furthermore, this approach of assigning an average value to a user would be limited for an area with a heterogeneous population. In that case, inference of socio-demographic attributes can be improved by drawing a value from a distribution instead of assigning an average value.

Finally, although our approach can extract useful information on user life-style choices, it has several limitations which include:

1. There could be demographic biases among social media users that can potentially impact our results. It is found that users of smartphones and location-based services have minor over representation from younger people [35].
2. There might be potential geographic biases if the users prefer to check-in from certain places in the city. In addition, we do not observe the complete sequence of the activities participated by an individual. These geographic biases and lack of activity sequences may ignore some important aspects of user life-style choices.

A few of these limitations may go away with wider uses of social media over the population. For instance, it is expected that with the wide-spread uses of location-based services in the future, sample representativeness of social media datasets will improve. None the less, given the available data, we are able to extract the geo life-style patterns and we anticipate that the observed patterns are not the exclusive traits of the social media users. Further research will be needed to account for missing observations and geographic biases.
